# From population to individuals: a new indicator for evaluating the appropriateness of clinical application of antibiotics

**DOI:** 10.1186/s40360-018-0245-y

**Published:** 2018-09-05

**Authors:** Bin Zheng, Na Li, Zhijian Hu, Maobai Liu

**Affiliations:** 1 Department of Pharmacy, Fujian Medical University Union Hospital, 29 Xinquan Road, Gulou District, Fuzhou City, 350001 Fujian Province China; 2 Department of Information, Fujian Medical University Union Hospital, 29 Xinquan Road, Gulou District, Fuzhou City, 350001 Fujian Province China

**Keywords:** Defined daily dose (DDD), Antibiotics, Ratio of use density to use rate (UD/UR), Appropriateness evaluation, Indicator

## Abstract

**Background:**

This study aims to establish a new indicator based on the anatomical therapeutic chemical/defined daily dose (ATC/DDD) system.

**Methods:**

Utilization data of antibiotics of inpatients in a university hospital were used to calculate the indicators of use rate (UR), use density (UD), and ratio of use density to use rate (UD/UR). According to the professional characteristics, the recommended values of UD/UR in different departments were established respectively. Crosswise comparison and appropriateness evaluation between different treatment groups with the same profession were performed. For individual inpatients with abnormally increased drug utilization index (DUI) and ratios of antimicrobial course to length of stay (C/S), detailed analysis was performed to examine whether any irrational drug utilization occurred.

**Results:**

The indicator UD/UR combines both dose and duration of treatment, which were the two main factors affecting the appropriateness of clinical application of antibiotics. Thus, it can more sensitively reveal the drug utilization of inpatients receiving antibiotics. UD/UR is also more suitable for evaluating the clinical appropriateness of antibiotic application than the macroscopic indicator, total UD, and could be applied at the macroscopic and microscopic levels.

**Conclusions:**

The ratio UD/UR has great practical value and can serve as a reference for evaluating the appropriateness of clinical application of antibiotics.

## Background

Irrational antibiotic utilization, the most important cause of the antibiotic resistance of bacteria, is a problem requiring effective supervision in the clinical application of antibiotics. The Anatomical Therapeutic Chemical (ATC) Classification System and the measurement unit of defined daily dose (DDD) [1] were recommended and officially regarded by the World Health Organization (WHO) as the international standard in drug utilization research (DUR) in 1996. The number of DDDs [2] and drug use density (DDDs per patient day [3], DDDs per 100 patient days [3–6], DDDs per 1000 patient days [7–10], DDDs per admission [3, 11], DDDs per 1000 admissions [3, 12, 13], or DDDs per operation [14]) established based on the ATC/DDD system have been widely used as important indicators of antibiotic utilization research. However, although they can be obtained easily and applied to continuously and systematically monitor the administration of antibiotics in a population, these macro-indicators are not meaningful in evaluating the appropriateness of drug utilization. In addition, establishing rigid standards is difficult. Consideration of the specific condition of each patient is required when micro-indicators such as indication, selection of variety, prophylactic administration time before operation, dose, frequency and course of administration are used for the appropriateness evaluation of clinical application of antibiotics. Unfortunately, in spite the high precision, strong expertise and long processing times are required to evaluate with these micro-indicators; thus, the feasibility of their application to bulk operation is poor. Similar to macro-indicators, establishing uniform standards for these micro-indicators is difficult [15]. Therefore, a more suitable indicator for the appropriateness evaluation of clinical application of antibiotics that could be adapted to both populations and individuals, must be developed. This article proposes a new indicator, i.e., ratio of use density to use rate (UD/UR), for the appropriateness evaluation of antibiotics and investigates its application at the macroscopic and microscopic levels.

## Methods

### Materials

The research materials included utilization data of antibiotics in a 2500-bed university hospital. The name, specification, dose, frequency, and course, among other information, of antibiotics used by inpatients in each department in a certain month were obtained from the Hospital Information System (HIS). All study protocols were performed in accordance with and approved by the Committee of Research Ethics of Fujian Medical University Union Hospital (2018KY013, Fuzhou, China).

### Methods

#### Derivation of equation

According to *Management of Medical Quality and Control Indicators of Tertiary General Hospitals* (version 2011) published by the National Health and Family Planning Commission (NHFPC) of the People’s Republic of China [16], the UR and UD of antibiotics used by inpatients in each department can be calculated using eqs. 1, 2, 3. Equation (4 can be derived from eqs. 1, 2, 3 to calculate the UD/UR in each department. Equation (9 can be derived from eq. 5 of the drug utilization index (DUI) and self-defined eqs. 6, 7, 8.

1 $$ \mathrm{UR}\ \left(\mathrm{proportion}\ \mathrm{of}\ \mathrm{inpatients}\ \mathrm{receiving}\ \mathrm{antibiotics}\right)\ \left(\%\right)=\mathrm{number}\ \mathrm{of}\ \mathrm{inpatients}\ \mathrm{receiving}\ \mathrm{antibiotics}/\mathrm{total}\ \mathrm{number}\ \mathrm{of}\ \mathrm{inpatients}\times 100\% $$

2 $$ \mathrm{UD}=\sum \left[\mathrm{consumption}\ \mathrm{of}\ \mathrm{antibiotics}\ \left(\mathrm{g}\right)/\mathrm{DDD}\ \left(\mathrm{g}/\mathrm{day}\right)\right]/\mathrm{number}\ \mathrm{of}\ \mathrm{patient}\ \mathrm{days}\times 100=\left(\mathrm{number}\ \mathrm{of}\ \mathrm{DDDs}\times 100\right)/\mathrm{number}\ \mathrm{of}\ \mathrm{patient}\ \mathrm{days} $$

3 $$ \mathrm{Number}\ \mathrm{of}\ \mathrm{patient}\ \mathrm{days}=\mathrm{total}\ \mathrm{number}\ \mathrm{of}\ \mathrm{inpatients}\times \mathrm{mean}\ \mathrm{length}\ \mathrm{of}\ \mathrm{stay}\ \mathrm{of}\ \mathrm{inpatients} $$

4 $$ \mathrm{UD}/\mathrm{UR}=\left[\mathrm{number}\ \mathrm{of}\ \mathrm{DDDs}\times 100\right]/\left[\mathrm{number}\ \mathrm{of}\ \mathrm{inpatients}\ \mathrm{receiving}\ \mathrm{antibiotics}\times \mathrm{mean}\ \mathrm{length}\ \mathrm{of}\ \mathrm{stay}\ \mathrm{of}\ \mathrm{inpatients}\right] $$

5 $$ \mathrm{DUI}=\mathrm{number}\ \mathrm{of}\ \mathrm{DDDs}/\mathrm{total}\ \mathrm{medication}\ \mathrm{days} $$

6 $$ \mathrm{Total}\ \mathrm{medication}\ \mathrm{days}=\mathrm{number}\ \mathrm{of}\ \mathrm{inpatients}\ \mathrm{receiving}\ \mathrm{antibiotics}\times \mathrm{mean}\ \mathrm{treatment}\ \mathrm{days}\ \mathrm{of}\ \mathrm{inpatients}\ \mathrm{receiving}\ \mathrm{antibiotics} $$

7 $$ \mathrm{Ratio}\ \mathrm{of}\ \mathrm{antimicrobial}\ \mathrm{course}\ \mathrm{to}\ \mathrm{length}\ \mathrm{of}\ \mathrm{stay}\ \left(\mathrm{C}/\mathrm{S}\right)=\mathrm{mean}\ \mathrm{treatment}\ \mathrm{days}\ \mathrm{of}\ \mathrm{inpatients}\ \mathrm{receiving}\ \mathrm{antibiotics}/\mathrm{mean}\ \mathrm{length}\ \mathrm{of}\ \mathrm{stay}\ \mathrm{of}\ \mathrm{inpatients}\ \mathrm{receiving}\ \mathrm{antibiotics} $$

8 $$ \mathrm{Elongation}\ \mathrm{factor}\ \mathrm{of}\ \mathrm{length}\ \mathrm{of}\ \mathrm{stay}\ \left(\mathrm{EF}\right)=\mathrm{mean}\ \mathrm{length}\ \mathrm{of}\ \mathrm{stay}\ \mathrm{of}\ \mathrm{inpatients}\ \mathrm{receiving}\ \mathrm{antibiotics}/\mathrm{mean}\ \mathrm{length}\ \mathrm{of}\ \mathrm{stay}\ \mathrm{of}\ \mathrm{inpatients} $$

9 $$ \mathrm{UD}/\mathrm{UR}=\mathrm{DUI}\times \left(\mathrm{C}/\mathrm{S}\right)\times \mathrm{EF}\times 100 $$

The UD, UR, and UD/UR of antibiotics of inpatients in each department were calculated. Based on the professional characteristics, the recommended values of UD/UR of some departments were established respectively.

The UD/UR of antibiotics of inpatients in different treatment groups with the same profession were calculated, and crosswise comparison and appropriateness evaluation were performed.

The UD/UR of the antibiotics of individual inpatients were calculated. Patients with abnormally increased DUI or C/S were identified, and their electronic medical records were studied to further examine whether any irrational drug utilization had occurred.

## Results

*The National Program of Special Renovation Activity on the Clinical Application of Antibiotics* published by the NHFPC in 2012 [17] states that “the UR should be no more than 60% and the UD should be controlled under 40 DDDs per 100 patient days in general hospitals”. In this research, the UD exceeded 40 DDDs in 17 departments and the UR exceeded 60% in 11 departments (Table 1). However, the UD were less than 40 DDDs and the UR exceeded 60% in both the geriatrics and gynecology departments. By contrast, the UD exceeded 40 DDDs and the UR were less than 60% in the departments of hematology, general surgery, nephrology, rheumatology, neurology, and burns. The UR and UD of antibiotics in these departments did not synchronously reach the recommended standards, thereby indicating that the results of the appropriateness evaluation of clinical application of antibiotics would be limited if such evaluation was performed using only these two indicators.

**Table 1:** Antibacterial utilization of inpatients in each department

Ranking	Department	UR	Department	UD	Department	UD/UR
1	Infectious diseases	100.00%	Infectious diseases	220.00	Hematology	348.04
2	Intensive care unit	88.89%	Respiratory care	170.33	Pediatric hematology	291.12
3	Respiratory care	81.19%	Intensive care unit	136.91	General surgery	249.30
4	Emergency surgery	79.35%	Emergency surgery	136.55	Oncology	224.47
5	Ear–nose–throat	77.50%	Hematology^b^	113.67	Infectious diseases	220.00
6	Emergency medicine	72.73%	Emergency medicine	101.52	Respiratory care	209.79
7	Neonatology	71.05%	Cardiac surgery	72.81	Neurology	197.49
8	Cardiac surgery	67.46%	Ear–nose–throat	67.99	Emergency surgery	172.09
9	Gynecology^a^	66.78%	Pediatric hematology	65.24	Neurosurgery	162.62
10	geriatrics ^a^	65.79%	General surgery^b^	64.32	Intensive care unit	154.02
11	Stomatology	64.96%	Stomatology	53.39	Interventional therapy	146.22
12	Urology	59.71%	Nephrology^b^	51.59	Pediatrics	143.01
13	Pediatric surgery	57.14%	Hepatobiliary surgery	51.57	Emergency medicine	139.58
14	Hepatobiliary surgery	54.47%	Pediatrics	49.48	Cardiology	136.92
15	Nephrology^b^	43.24%	Rheumatology^b^	44.7	Thyroid gland surgery	129.55
16	Burns^b^	41.67%	Neurology^b^	40.88	Rheumatology	124.17
17	Orthopedics	40.91%	Burns^b^	40.47	Endocrinology	123.20
18	Gastroenterology	38.92%	Urology	37.62	Nephrology	119.31
19	Rheumatology^b^	36.00%	Neurosurgery	35.89	Colorectal surgery	114.77
20	Thoracic surgery	35.58%	Thoracic surgery	34.63	Cardiac surgery	107.93
21	Pediatrics	34.60%	Pediatric surgery	33.52	Breast surgery	103.33
22	Hematology^b^	32.66%	geriatrics ^a^	31.39	Thoracic surgery	97.33
23	Obstetrics	32.34%	Neonatology	31.06	Burns	97.12
24	Gastrointestinal surgery	30.60%	Colorectal surgery	30.38	Hepatobiliary surgery	94.68
25	Colorectal surgery	26.47%	Gastroenterology	29.78	Ear–nose–throat	87.73
26	General surgery^b^	25.80%	Orthopedics	27.71	Traditional Chinese medicine	87.35
27	Radio-oncology	23.57%	Cardiology	26.11	Stomatology	82.19
28	Pediatric hematology	22.41%	Gynecology^a^	25.39	Gastrointestinal surgery	81.90
29	Neurosurgery	22.07%	Gastrointestinal surgery	25.06	Gastroenterology	76.52
30	Traditional Chinese medicine	21.74%	Endocrinology	21.19	Orthopedics	67.73
31	Neurology^b^	20.70%	Oncology	19.08	Radio-oncology	65.21
32	Cardiology	19.07%	Traditional Chinese medicine	18.99	Urology	63.00
33	Plastic surgery	18.86%	Radio-oncology	15.37	Pediatric surgery	58.66
34	Endocrinology	17.20%	Obstetrics	13.81	Rehabilitation	57.61
35	Day care ward	12.15%	Interventional treatment	8.13	Day care ward	57.37
36	Oncology	8.50%	Plastic surgery	7.88	geriatrics	47.71
37	Rehabilitation	7.69%	Day care ward	6.97	Neonatology	43.72
38	Intervention treatment	5.56%	Thyroid gland surgery	5.48	Obstetrics	42.70
39	Thyroid gland surgery	4.23%	Rehabilitation	4.43	Plastic surgery	41.78
40	Ophthalmology	1.31%	Breast surgery	0.93	Ophthalmology	38.93
41	Breast surgery	0.90%	Ophthalmology	0.51	Gynecology	38.02

^a^UD was < 40 DDDs, whereas UR was > 60%

^b^UR was < 60%, whereas UD was > 40 DDDs

According to eq. 9, given that it was positively correlated with the product of DUI, C/S, and EF, UD/UR could be decomposed into three terms for individual analysis. DUI is usually applied as a standard to judge the appropriateness of clinical medication [18, 19]. For a single variety of antibiotics, DUI > 1.0 means the prescribed daily dose (PDD) of the drug is more than the DDD, indicating the possibility of overdose. DUI ≤ 1.0 means the PDD of the drug is less than or equal to its DDD, indicating that the dose is rational [20]. Hence, different recommended values of DUI can be established according to the professional characteristics of each department. For departments such as hematology, respiratory care, or intensive care unit, inpatients are usually prescribed with therapeutic purpose and receive combined utilization of two to three types of different antibiotics; thus, the rational value of DUI can be assigned as 2 or 3. For departments such as thyroid gland surgery or breast surgery, inpatients are usually prescribed with a single type of prophylactic antibiotics; thus, the rational value of DUI can be assigned as 1. Different recommended values of C/S can also be established according to the professional characteristics of each department. For therapeutic departments, such as hematology, respiratory care, or intensive care unit, antimicrobial courses usually last from admission to hospital discharge; thus, the rational ratio can be set as 1. For preventative departments, such as thyroid gland surgery or breast surgery, the duration of prophylactic antibiotics is usually less than or equal to 1 day (24 h); thus, the rational ratio can be set as “one day to the mean length of stay of inpatients receiving antibiotics”. The EF reflects the extension degree of the mean length of stay of inpatients receiving antibiotics compared with all inpatients and its rational value is usually set as 1. Considering DUI, C/S, and EF, the recommended values of UD/UR of antibiotics of inpatients in some departments can be obtained (Table 2).

**Table 2:** Recommended values of UD/UR of antibiotics of inpatients in some departments

Department		Recommended values			
		DUI	C/S	EF	UD/UR
Therapeutic department	Hematology	3	1	1	300
	Respiratory care	2	1	1	200
	Intensive care unit	2	1	1	200
Preventative department	Thyroid gland surgery	1	1/(9.15^a^)	1	10.93
	Breast surgery	1	1/(5.77^b^)	1	17.33

^a^Mean length of stay of inpatients receiving antibiotics in the department of thyroid gland surgery

^b^Mean length of stay of inpatients receiving antibiotics in the department of breast surgery

In Table 1, the UD/UR of departments of thyroid surgery and breast surgery were > 100, indicating a high possibility of irrational medication and requirement of enhanced monitoring. However, the UR and UD of antibiotics in these two departments reached the recommended standards with extremely low values. This finding reveals that UD/UR has high sensitivity in the appropriateness evaluation of clinical antibiotics application. UD/UR was > 300 in the department of hematology and were > 200 in the departments of general surgery, oncology, infectious diseases, and respiratory care, which indicates a possibility of irrational medication and requirement of enhanced monitoring.

In Table 3, crosswise comparison revealed that UD/UR was obviously higher in group G of the oncology department, group I of the hematology department, group A of the respiratory care department, group B of the rheumatology department, group A of the cardiology department, group C of the neurology department, group A of the endocrinology department, group A of the ophthalmology department, group A of the gastrointestinal surgery department, group A of the neurosurgery department, group C of the burn department, group C of the stomatology department, group C of the hepatobiliary surgery department, group B of the cardiac surgery department, group B of the interventional treatment department, and group C of the breast surgery department than in other groups with the same profession. Thus, enhanced monitoring is required in these groups.

**Table 3:** UD/UR of antibiotics of inpatients in each treatment group of some departments

Department	Group A	Group B	Group C	Group D	Group E	Group F	Group G	Group H	Group I	Group J
Oncology	199.39	99.96	58.18	345.43	10.81	372.64	437.28	0.00		
Hematology	252.42	487.37	274.90	183.90	228.55	304.81	333.36	307.78	519.13	190.67
Respiratory care	269.39	174.31	192.60	172.77						
Rheumatology	114.60	331.25	197.56							
Cardiology	143.28	37.17	52.75	136.19	48.45	124.87	85.10			
Neurology	129.81	85.27	157.10							
Endocrinology	137.44	98.13	81.61	121.60						
Ophthalmology	113.39	14.51								
Gastrointestinal surgery	139.02	40.55	48.93							
Neurosurgery	259.62	138.40	68.47	127.31	215.94	15.79				
Burns	38.23	104.79	123.05							
Stomatology	35.29	51.00	111.80							
Hepatobiliary surgery	89.35	127.85	156.00	52.62						
Cardiac surgery	37.22	193.25	129.98	54.78	78.90	131.08	49.34	69.98	54.62	65.98
Interventional treatment	0.00	139.23	71.59							
Breast surgery	0.00	38.41	459.63	227.82						

UD/UR was > 300 in groups B, F, G, H, and I of the hematology department; It was > 200 in groups D, F, and G of the oncology department; group A of the respiratory care department; and group B of the rheumatology department; It was > 100 in groups A, D, and F of the cardiology department; group A of the ophthalmology department; group A of the gastrointestinal surgery department; groups A, B, D, and E of the neurosurgery department; group C of the stomatology department; group B of the interventional treatment department; and groups C and D of the breast surgery department. These findings indicate a possibility of severe infection or irrational medication. Hence, enhanced monitoring is required in these groups.

For individual inpatients, UR = 100% and EF = 1. eq. (9) can be written as UD = DUI × (C/S) × 100 (10). Based on this equation, the brief chart in Table 4 was designed in the HIS in coordination with the information department of the hospital. Autonomous statistics can be achieved at any time to identify patients with abnormally increased DUI and C/S. In addition, the brief chart can be linked to a patient’s electronic medical records, thereby enabling detailed analysis on the rationality of medication.

**Table 4:** Brief chart of antibacterial utilization of individual inpatients

Record number	Treatment group	Treatment days	Length of stay	DDDs	DUI	C/S	UD
78 × ×89	Group × of neurosurgery department	3.00	3.00	4.50	1.50	1.00	150.00
77 × ×74	Group × of stomatology department	3.00	6.00	6.00	2.00	0.50	100.00
78 × ×09	Group × of urology department	1.00	12.00	2.00	2.00	0.08	16.67

For example, DUI of patient 78 × × 89 was relatively high. Review of the patient’s electronic medical records showed subarachnoid hemorrhage and aneurysm of the posterior communicating artery. On January 24, intracranial aneurysm clipping was performed. Ceftriaxone was administered at a dosage of 3 g per day (qd) through intravenous infusion (ivgtt) for surgical prophylaxis from January 24 to January 26. The *Indications and Usage* section [21] specifies that for surgical prophylaxis, a single 1 g-dose of ceftriaxone administered ivgtt 0.5–2 h before operation is recommended. Thus, the dose of 3 g qd was too high in this case.

C/S of patient 77 × × 74 was also relatively high. Review of the patient’s electronic medical records showed that the patient underwent open reduction and internal fixation for a complex mandibular fracture on January 3. A dose of 2 g of cefathiamidine was offered three times per day (tid) through ivgtt from January 3 to January 5. The duration of prophylactic antibiotics applied to this operation should be no more than 24 h [22]. However, the antimicrobial course of this case was 72 h, which was excessively long.

C/S of patient 78 × × 09 was > 0. Review of the patient’s electronic medical records showed treatment by hernioplasty for an inguinal hernia on January 15 and administration of 2 g of cefathiamidine tid through ivgtt on that day. However, prophylactic administration of antibiotics is not recommended for such an operation [23, 24]. Thus, application of preventative antibiotics was irrational in this case.

## Discussion

DDD is the most commonly used measurement unit recommended by the WHO for DUR [25–28], and several studies have focused on its differentiation in the clinical setting. Kritsotakis et al. [29] stratified the UR and UD of antibiotics according to clinical service, which allowed areas of concern to be specified and targeted antibiotic policy changes to be initiated. A number of disease-specific quality indicators have also been established. For instance, the range of UR of antibiotics for acute cystitis should be 80–100% while that of quinolones should be 0–5%, as recommended by the European Surveillance of Antimicrobial Consumption [30]. Some researchers have investigated other indicators, such as the ratio of number of DDD to number of treatment days [5], daily doses per admission (DDDs per admission) [12, 31], and number of days of therapy per 1000 patient days (DOTs per 1000 patient days) [32]. While undoubtedly useful, all of these indicators place emphasis on the difference between administered dosage (prescribed daily doses, PDD) and DDD and ignore the bias of length of stay of different inpatients.

The indicators UR and UD could be suitable for representing antibacterial utilization at the macroscopic level. UR reflects the administration proportion while UD reflects the administration density in a population. When UR remains unchanged, the utilization condition in a population is codetermined by the individual utilization condition of every inpatient. By deriving formulas and extending the meanings of some indicators, such as DUI, the indicator UD/UR was innovatively presented in this article based on the ATC/DDD system. UD/UR synchronously considers the dose and duration of treatment, the two main factors affecting the appropriateness of clinical application of antibiotics, and can sensitively reflect the drug utilization of inpatients receiving antibiotics. Combined with the indicators UR and UD, the proposed indicator could be more suitable for evaluating the clinical appropriateness of antibiotics application than total UD.

UD/UR can be applied to the real-time monitoring, comparison, and evaluation of the appropriateness level of antibiotics utilization in hospitals, departments, treatment groups, and individual patients at the macroscopic and microscopic levels. For example, the control goals of antibiotics can be established in different departments at the department level, utilization levels of antibiotics in different treatment groups with the same profession can be compared at the treatment-group level, and abnormal utilization condition can be detected to provide an early warning at the individual-patient level. Various indicators, including UD, UR, and UD/UR, should be considered synthetically when performing the appropriateness evaluation of clinical application of antibiotics to address the limitations of each indicator and produce comprehensive, authentic, precise, and fair evaluation results.

Individualized setting and continuous improvement of the recommended values of UD/UR in clinical practice should be enabled based on the different professional characteristics of various departments or hospitals, such as distribution of disease entities, medication regimens, and mean length of stay. For example, if only one dose of preventative antibiotics (assuming that the frequency of administration is tid) can be used during the period of some operations with type I (clean) incisions, the rational DUI can be assigned as (1/3)/(1/3) = 1 and C/S as [(1/3)/(mean length of stay of inpatients receiving antibiotics)]. If no preventative antibiotics can be used during the period of some operations with type I (clean) incisions in principle, the rational DUI and C/S can both be assigned as 0. The implications of the indicator UD/UR make it extremely suitable for managing of clinical pathways and charge based on disease entities (diagnosis-related groups, DRGs). It is also useful for the specification and stepwise implementation of the responsibility system of clinical application management of antibiotics.

## Conclusions

UD/UR can sensitively reflect the drug utilization of inpatients receiving antibiotics and be applied at the macroscopic and microscopic levels. This indicator has great practical value and can serve as a reference for evaluating the appropriateness of clinical application of antibiotics.

## Abbreviations

C/S: ratio of antimicrobial course to length of stay
DDD: Defined daily dose
DUI: Drug utilization index
EF: Elongation factor of length of stay
PDD: Prescribed daily dose
UD: Use density
UD/UR: Ratio of use density to use rate
UR: Use rate
